# CryoEM milestones and future directions

**DOI:** 10.1063/4.0001045

**Published:** 2025-10-27

**Authors:** Tamir Gonen

**Affiliations:** UCLA, Los Angeles, ca, USA

## Abstract

In this seminar, I will provide a comprehensive overview of the transformation of cryo-electron microscopy (cryo-EM) from a technique once characterized by low-resolution "blobology" to a powerful tool that has revolutionized structural biology. Cryo-EM has enabled the visualization of biological macromolecules at near-atomic resolution, providing unprecedented insights into molecular mechanisms and expanding the boundaries of structural biology beyond what was possible with traditional methods such as X-ray crystallography and NMR spectroscopy.

I will discuss the various modalities of cryo-EM, including single-particle analysis, cryo-electron tomography (cryo-ET), and MicroED, highlighting the key technological and methodological breakthroughs that have propelled the field forward. The seminar will trace the historical development of cryo-EM, beginning with early ultrastructure studies using freeze-fracture methods, followed by the advent of vitrification, which allowed for the preservation of biological specimens in a near-native state. I will then explore the transformative impact of direct electron detectors and advanced image processing algorithms, which have significantly enhanced resolution and data interpretation capabilities.

A particular focus will be given to MicroED (microcrystal electron diffraction), a technique that straddles both crystallography and electron microscopy, offering a powerful approach for solving structures of small and challenging macromolecular crystals. I will discuss its applications in drug discovery, small-molecule structure determination, and the study of protein-ligand interactions, as well as recent advancements in automation and data collection strategies that are making MicroED more accessible to the broader scientific community.

Through this seminar, I aim to provide an appreciation of how cryo-EM has evolved into a cornerstone of modern structural biology, its impact on fundamental biological discoveries, and its potential for future innovations.

